# The DNA Damage Response Induced by Infection with Human Cytomegalovirus and Other Viruses

**DOI:** 10.3390/v6052155

**Published:** 2014-05-23

**Authors:** Xiaofei E, Timothy F. Kowalik

**Affiliations:** 1 Department of Microbiology and Physiological Systems, University of Massachusetts Medical School, 368 Plantation St. Worcester, MA 01605, USA; E-Mail: xiaofei.e@umassmed.edu (X.E); 2 Program in Immunology and Microbiology, University of Massachusetts Medical School, 368 Plantation St, Worcester, MA 01605, USA

**Keywords:** HCMV, cell cycle, DNA damage response, DDR, ATM

## Abstract

Viruses use different strategies to overcome the host defense system. Recent studies have shown that viruses can induce DNA damage response (DDR). Many of these viruses use DDR signaling to benefit their replication, while other viruses block or inactivate DDR signaling. This review focuses on the effects of DDR and DNA repair on human cytomegalovirus (HCMV) replication. Here, we review the DDR induced by HCMV infection and its similarities and differences to DDR induced by other viruses. As DDR signaling pathways are critical for the replication of many viruses, blocking these pathways may represent novel therapeutic opportunities for the treatment of certain infectious diseases. Lastly, future perspectives in the field are discussed.

## 1. Introduction

Human cytomegalovirus (HCMV) is a β-herpesvirus and is genetically the most complex viral pathogen of humans. Though HCMV infection rarely causes symptomatic disease in immunocompetent individuals, it can establish lifelong latency/persistence following primary infection and can be reactivated under some conditions. In general, it is the causative agent of a variety of disorders in immunocompromised and immunosuppressed individuals. HCMV-associated pneumonitis and retinitis are among the most prevalent complications following primary infection or reactivation of latent HCMV reservoirs. HCMV infections are serious threats to the health of HIV-positive individuals and transplant patients. Primary or reactivated HCMV infections can place pregnancies at risk as the virus can be transmitted to fetuses. In the United States, about 1% of newborns are congenitally infected with HCMV. Most congenitally infected infants and children do not present with health problems, but 22%–38% of infected infants are born with symptoms, including microcephaly and mental retardation [1,2,3]. HCMV is also the leading cause of nonfamilial hearing loss. A strong association between HCMV and glioblastomas has been established [4], but its direct role in tumorigenesis is, at this juncture, unclear.

Many environmental factors and physiological processes can damage DNA. The presence of abnormal DNA structures can induce many types of DNA signaling pathways. Mammalian viruses use different strategies to antagonize host defense systems, including altering host DNA damage response (DDR) to facilitate their replication. This review concentrates on the effects of DDR on HCMV replication and draws comparison to other viruses that induce DDR. 

## 2. HCMV

HCMV virions are structurally complex. It is an enveloped DNA virus that contains a dsDNA genome of ~235 kilobase pairs, which is the largest genome of any human virus [5]. The genome encodes approximately 200 open reading frames [6,7] although a recent study suggests that many additional, small open reading frames are also transcribed and translated [8]. Recent study shows that HCMV genomes exists as complex mixtures of variants in patients, which may add another layer of genetic complexity to viral infections [9]. The viral genome is encased within a capsid and surrounded by a protein layer called the tegument [10,11]. This structure contains proteins that are delivered to cells upon infection and can act before the onset of viral immediate early (IE) gene expression to help initiate a productive infection. As examples of tegument proteins with such activity, pp71, promotes the degradation of hypophosphorylated forms of pRB, p107, and p130, thus stimulating activities associated with cell cycle progression, whereas tegument delivered pUL69 arrests the cell cycle in a late G_1_/S-like state [12].

Productive HCMV replication and gene expression has been subdivided into three kinetic classes: immediate early (IE), early (E), and late (L) [13,14]. IE genes are the first to be expressed and do not require *de novo* protein synthesis for their expression. The IE proteins have many functions which collectively prepare the host cell and viral genome for E gene expression and viral DNA replication. In general terms, E gene products are associated with promoting viral DNA replication. The replication of viral DNA is closely associated with expression of L genes. IE and E gene products also regulate late gene expression [15]. The 72-kDa IE1 protein and 86-kDa IE2 proteins are the first and, for IE1 at least, among the most abundantly expressed proteins during HCMV infection. Both proteins have long been recognized as transcriptional regulators, but they also interact with numerous cellular proteins including RB family members [16,17]. They are produced from differentially spliced transcripts under the control of strong promoter-enhancer element known as the major immediate early promoter (MIEP).

HCMV early genes require prior *de novo* synthesis of viral IE and cellular proteins for their transcription. The earliest of the early gene transcripts appear and accumulate to peak levels by 8 hours postinfection (e.g., UL112–113), while temporally later early transcripts can be detected just prior to the onset of viral DNA replication (e.g.,TRL4) and accumulate to peak levels when viral DNA replication is allowed to proceed [18]. Most of the viral early genes function in one of two ways. Some of the early genes are directly involve in viral DNA synthesis, cleavage and packaging of the viral genome, and contribute to assembly of the virus particles. Some other genes function to produce cellular and extracellular environments that are suitable for viral gene expression and replication, either by modulating factors involved in the regulation of cellular DNA synthesis or by altering the host’s immune response to the virus. Some examples of the early genes are the UL112-113 nuclear phosphoproteins and HCMV viral DNA replication proteins, including DNA polymerase processivity factor (UL44) and single-stranded DNA binding protein (SSB) (UL57), which are localized in nuclear replication compartments [19].

Following viral DNA replication, delayed early and late viral genes are expressed which, in general, encode the structural components of the virion. UL55 (gB), UL75 (gH) and UL99 (pp28) as well as components of the capsid are products of late genes. While much study has been carried on the regulation of HCMV IE and E gene expression, little is known about the specific mechanisms of regulating late gene expression.

A key biological property of HCMV is to maintain a lifelong relationship with its host by way of latent or persistent infections. During latency, only a subset of viral genes is expressed. The mechanisms governing the establishment and maintenance of latency and reactivation of HCMV from latency are complex and now coming into focus. HCMV resides latently in hematopoietic cells of the bone marrow. Several *in vitro* systems have been developed as models for HCMV latency. Nelson and colleagues [20,21] have used allogenic stimulation to study HCMV reactivation in monocytes that harbor viral genomes. CD4+ and CD8+ T lymphocytes, cytokines, IFN-γ, and tumor necrosis factor-α can facilitate viral reactivation [21,22]. Mocarski and colleagues [23,24] have studied HCMV latency in granulocyte–macrophage progenitors expressing CD33 and dendritic cell markers. They have identified several HCMV transcripts expressed during latency following *in vivo* or *in vitro* infection. Goodrum and colleagues have investigated a primary CD34 (+) hematopoietic progenitor cell system as an experimental model to study HCMV latency and reactivation [25]. Using an HCMV gene array, they examined HCMV gene expression in these cells. CD34+ cells exhibit distinct patterns of viral gene expression from that observed during productive or nonproductive infections. Furthermore, pUL138 was identified as an HCMV protein that promotes an infection with the hallmarks of latency [26,27]. Sinclair and colleagues analyzed the secretome of cells carrying latent HCMV and have identified changes in several secreted cellular proteins known to be involved in regulation of the immune response and chemoattraction [28]. Their results identified a strategy by which sites of latent HCMV can firstly recruit CD4+ T cells and then inhibit their antiviral effector functions. All told, much more needs to be learned in order to develop a clear understanding of HCMV latency.

In sum, HCMV infection strategies and viral replication are complex and reflective of the large genome, numbers of proteins (and miRNAs), and broad cell tropism. 

## 3. Cell Cycle Checkpoints

Cell cycle checkpoints are regulatory steps in pathways that govern the order and timing of cell cycle transitions to ensure completion of one cellular event prior to commencement of another. Checkpoints also offer the opportunity to repair damaged DNA. Most eukaryotic cells proceed through an ordered cell cycle, G1→S→G2→M phase, during which the chromosomes and other cell material double in number with each of the daughter cells receiving one copy of the doubled genetic material. The cell cycle is completed when each daughter cell has its own intact outer membrane. Regulation of the cell cycle is the key for the normal development of multi cellular organisms.

In the cell cycle, the G1 phase represents an organizing state prior to DNA replication and where decisions regarding cell cycle progress are made. Factors that influence progression through G1 include cell size, metabolic state, cell signaling, and perhaps a need to repair damaged DNA. Inconsistency among these states or excessive DNA damage can lead cells to exit the cell cycle and undergo senescence or apoptosis. S phase is where DNA synthesis takes place resulting in the duplication of the cellular genome. In the G2 phase, the cell prepares for the process of mitosis, and the associated cell division to form two daughter cells. This stage provides another opportunity for recognition and repair of damaged DNA. Thus, under normal conditions, the progression of DNA replication and mitosis is signaled by the intracellular checkpoints primarily at the G1 and G2, respectively.

## 4. DNA Damage Response (DDR)

Many external and internal effectors, such as ionizing radiation and reactive oxygen species, can directly damage DNA. The presence of abnormal DNA structures, including single-stranded break, double-strand breaks, modification of incorporated nucleotides or aberrant replication fork structures, as well as alterations in higher-order chromatin structure, can induce one or more DDR. For a more complete review of DNA damage and responses, please refer to these publications [29,30,31,32,33].

**Figure 1:**
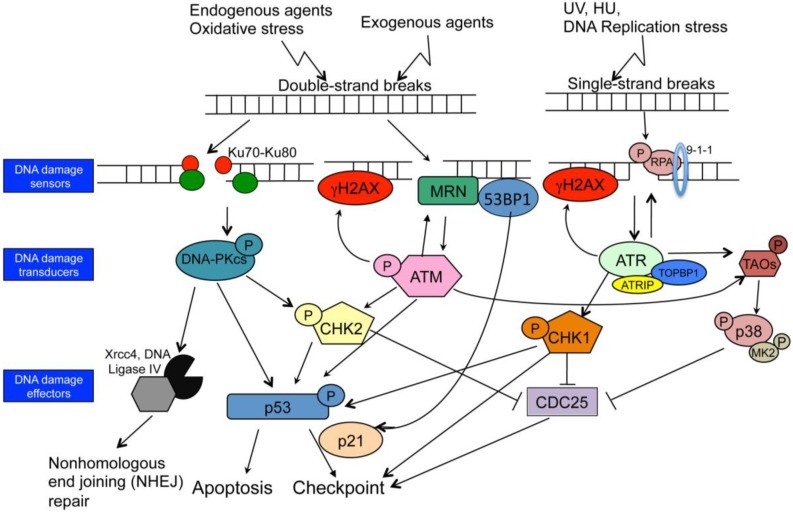
DNA damage-induced cell cycle checkpoint network. Schematic representation of ATM, ATR and DNA-PK signaling pathways. DNA-PK responds to DNA double-strand breaks and regulates nonhomologous end joining (NHEJ). The DNA ends are captured by the KU heterodimer. Ku regulatory proteins recruit DNA-PK to double-strand breaks; two DNA-PKcs molecules in concert tether DNA ends together and recruit the DNA Ligase IV–XRCC4 complex to rejoin broken DNA ends. ATM responds to DNA double-strand breaks; phosphorylates H2AX and NBS1, which localize to sites of DNA damage, where upon the MRN complexes form. ATM activation regulates cell-cycle checkpoints through the phosphorylation of CHK2 and p53. ATR is activated in response to single-stranded DNA (ssDNA). Activation of ATR requires TopBP1. ATR is recruited to RPA-coated ssDNA by its binding partner ATRIP. ATR regulates the cell-cycle through activation of CHK1. Both ATM and ATR are required to activate the p38MAPK/MK2 effector kinase complex downstream of TAO kinases in response to DNA damage. The three effector kinases, CHK1, CHK2, and MK2 are directly responsible for inhibitory phosphorylation on members of the Cdc25 family. Arrows indicate the flow of the respective DDR pathways.

Activation of the DDR plays a key role in avoiding or reducing errors. Although cells use different signaling pathways (Figure 1) to deal with different environment stresses, there are common elements. Signaling networks in response to DNA damage consists of sensors, transducers, and effectors. Sensors detect damaged DNA and signal to transducers. Transducers amplify and transfer the signal to effectors. Effectors then execute the cellular response to initiate cell cycle checkpoint activation, DNA repair or apoptosis. Cellular responses to DNA damage are crucial for maintaining genome integrity. Defects in the DDR system are also associated with several inherited human disorders [34,35,36] and cancers [37,38]. The DDR system tends to be more error prone than genomic replication and any remaining damage or incorrectly repaired damage may play a role in the development of pathologies such as birth defects, cancer or aging.

DNA lesions trigger the activation of various kinases, which play important roles in DDR (Figure 1). The phosphatidylinositol-3*-*kinase-like family, including ataxia-telangiectasia mutated (ATM), ataxia-telangiectasia Rad3-related (ATR) and DNA*-*dependent protein kinase catalytic subunit (DNA-PKcs) play central roles in DNA damage checkpoints. ATM is defective in ataxia-telangiectasia mutated disease (A–T), which is characterized by cancer susceptibility, radiosensitivity and neurological defects [39]. ATM is the primary mediator of the response to DNA double strand breaks (DSBs); ATM has been traditionally considered a nuclear protein that functions in response to genotoxic damage though ATM also participates in the oxidative stress response and cytoplasmic signaling [40,41,42,43,44]. ATR activation is generally associated with single-stranded DNA breaks and stalled DNA replication forks. DNA-Pkcs is an important enzyme involved in the non-homologous-end-joining pathway of double strand break repair [45]. The phosphorylation of these proteins plays a crucial role in the activation of various effector proteins. A large-scale proteomic study on ATM and ATR substrates identified more than 700 proteins that are phosphorylated in response to DNA damage [46].

## 5. The pRB-E2F Complex

The RB protein family consists of three members—pRB, p107, and p130—which maintain cells in a quiescent state as well as regulate the transition from G0/G1- to S-phase by modulating the activity of the E2F family of transcription factors. E2F transcription factors are the major downstream targets of the RB family of proteins, and are necessary for the expression of many genes that are required for cell cycle progression [47,48,49,50]. RB family members exert their growth regulatory functions partly by inhibiting the transcriptional activity of E2F [51,52,53,54,55]. Conversely, disruption of the *RB* gene by deletion or mutation, or inactivation of pRB by phosphorylation or interaction with viral oncoproteins, cause the release of free or now depressed and transcriptionally active E2F1-3 resulting in cell cycle progression [56,57,58]. Sustained inactivation of pRB often results in apoptosis or deregulated proliferation.

The E2F family, including E2F1-8, can be divided into two subgroups based on their primary function, activator E2Fs: E2F1, 2 and E2F3a, and suppressor E2Fs: E2F3b, E2F4-8. E2Fs 1–3a are required for the transactivation of target genes involved in the G_1_/S transition. E2F3 encodes two proteins, E2F3a and E2F3b that differ in expression pattern and function. E2F3a is a transcriptional activator mainly expressed during S phase, while E2F3b acts as a transcriptional repressor, which is constantly expressed during cell cycle. In contrast, E2F4 and E2F5 possess predominantly repressive activity. E2F4 and E2F5 bind to p107 and p130 with high affinity, and recent studies have demonstrated that they also interact with pRB [59,60,61,62,63]. E2F6 and E2F7 are also considered to be transcriptional repressors [64,65,66,67,68,69]. E2F6–8 are distinct from the other E2Fs in that they do not bind to pocket proteins. E2F6 is known to interact and form complexes with the Polycomb Group (PcG) proteins. It is not yet clear what the identity is of the interacting partners of these E2Fs. Deregulation of the pRB-E2F interaction results in hyperproliferation, lack of differentiation, genomic instability and can lead to cancer.

## 6. The Link between Cell Cycle and DDR

### 6.1. CHK1 and CHK2 Kinases Control the Cell Cycle in Response to DNA Damage

DNA damage poses a continuous threat to genomic integrity in mammalian cells. In order to prevent the propagation of damaged DNA through the cell cycle, cells have evolved DDR that coordinate cell cycle progression and checkpoints with the repair of DNA lesions. Mammalian cells initiate cell cycle arrest at different phases of the cell cycle in response to various forms of genotoxic stress to allow time for DNA repair. Cell cycle arrest and apoptosis are two of the downstream consequences of DDR [70]. CHK1 and CHK2 are checkpoint kinases that are activated by ATM or ATR and phosphorylate cell-cycle components to cause the arrest of the cell cycle [71,72] (Figure 1). Although CHK1 and CHK2 have overlapping roles, CHK1 kinase is restricted to S and G2 where its activity is amplified in the presence of different types of DNA damage [73]. CHK2 is expressed throughout the cell cycle and is also activated in the presence of DNA damage [74]. Usually, CHK1 or CHK2 phosphorylated p53 and CDC25 propagate signals to arrest cells or to undergo apoptosis depending on cell type and extent of DNA damage. However, DNA lesions sometimes do not induce cell cycle checkpoint responses, such as DNA damage during G2 phase of the green alga, *Scenedesmus quadricauda* [75]; or the level of DNA damage is low enough that the cell can deal with the lesions in the absence of a checkpoint response. 

### 6.2. A Novel Cell Cycle Checkpoint Kinase Pathway, MK2, that also Induced Cell Cycle Arrest

Over the last decade, a number of publications point to a crucial role for the p38MAPK/MAPKAP-K2 (MK2) complex as an integral part of the DDR network [76,77]. There are four p38MAPK isoforms denoted α, β, γ and δ [78]. p38α and p38β have been shown to be activated by DNA damage-specific agents, such as cisplatin, doxorubicin, and temozolomiode [76,77,79,80,81]. p38α forms a nuclear complex with its downstream substrate MK2 and that upon activation of p38MAPK in this complex, p38MAPK phosphorylates and activates MK2. p38MAPK/MK2 complex is a third checkpoint effect or module that operates parallel to CHK1 and is activated downstream of ATM and ATR [76,77,82] (Figure 1). More detailed information on this DNA damage checkpoint signaling pathways can be found elsewhere [82,83,84]. 

## 7. Many Viruses can Induce DNA Damage Responses and Modulate Cell Cycle Progression

DDR can be activated not only by external sources of DNA damage, but also by intracellular conditions, such as oncogene overexpression, loss of tumor suppressors, and viral infections. Recent studies demonstrate that infections by DNA viruses or viruses with a DNA genome stage during infection induce host DDR (Table 1) [85,86,87,88,89]. Somewhat unexpectedly, some viruses with RNA genomes can also induce DDR. The mechanisms responsible for DDR induction by RNA viruses are less clear and possibly indirect. 

The manipulation of the cell cycle by viruses is closely related to activation of the DDR and is usually associated with DNA double-strand break signaling pathways. As examples, oncoproteins of DNA tumor viruses, such as adenovirus E1A, simian virus 40 T antigen, and papillomavirus E7, each interact with the RB family of tumor suppressors, leading to E2F-mediated cell cycle stimulation, apoptosis induction, and cellular transformation. These proteins utilize a conserved LXCXE motif, which is also found in cellular proteins, to target the RB family. The subsequent induction of cell cycle checkpoints and activation of ATM/ATR/DNAPKcs pathways have been reported to accompany infection by a number of different viruses. Given that DDR usually results in cell cycle checkpoints and apoptosis, infection-associated DDR were initially considered to be antiviral, as in the case of adenovirus serotype 5 (Ad5) [90]. However, for many other viruses that induce a host DDR, the response appears to modulate the cell cycle at a precise point that favors virus replication [91,92,93]. Thus, manipulation of the cell cycle and the associated DDR may be a commonly employed strategy of viruses to create a favorable cellular environment for replication. 

Several mammalian viruses evolved mechanisms to manipulate DDR pathways for their own benefit by exploiting or actively inhibiting different parts of the pathways [91]. For example, simian virus type 40 (SV40), herpes simplex virus 1(HSV-1), HCMV, and Epstein-Barr virus (EBV) all activate ATM and downstream signaling during infection, which is accompanied by recruitment of ATM and other repair proteins to sites of viral DNA replication [94,95,96,97] (Table 1).

**Table 1:** A list of viruses that both induce and require host DNA damage responses (DDR) for productive infections.

Virus that Induce DNA damage response (DDR)	Abbreviation	Virus type	DDR factors activated	DDR factors required for virus replication	References
Human cytomegalovirus	HCMV	dsDNA, β-herpesvirus	ATM, CHK2, p53, H2AX NBS1, CHK1	ATM, p53, H2AX	[94,98]
Herpes simplex virus type 1	HSV-1	dsDNA, α-herpesvirus	ATM, CHK2, 53BP1, NBS1	ATM, Mre11	[95]
Epstein-Barr virus	EBV	dsDNA, γ-herpesvirus	ATM, CHK2, Nbs1, H2AX, p53, CHK1	XPC	[96,99]
Murine gammaherpesvirus 68	γHV68	dsDNA, γ-herpesvirus	ATM, H2AX, p53, CHK1	ATM, H2AX	[100,101]
Simian virus type 40	SV40	dsDNA, polyomavirus	ATM, CHK1, CHK2, p53	ATM, Rad51, FancD2	[97,102,103]
Human papillomavirus	HPV	dsDNA, papillomavirus	ATM, CHK2, H2AX, NBS1, CHK1, BRCA1	ATM, CHK2	[104,105,106,107,108]
Human parvovirus B19	B19V	ssDNA, parvovirus	ATM, CHK2, ATR, DNA-PKcs, CHK1, Ku70/Ku80, H2AX, RPA-32	ATR, CHK1, DNA-PKcs, Ku70/ku80	[109,110,111]
Adeno-associated virus	AAV	ssDNA, parvovirus	ATM, CHK2, DNA-PKcs, SMC1, H2AX, CHK1, RPA32	DNA-Pkcs	[112,113]
Human T-cell lymphotrophic virus type 1	HTLV1	ssRNA/dsDNA, retrovirus	ATM, CHK2, H2AX, NBS1, DNA-PKcs	N/A	[114,115,116,117,118,119]
Human immunodeficiency virus type 1	HIV-1	ssRNA/dsDNA, lentivirus	ATM, H2AX, p53, NBS1, ATR, CHK1, P38MAPK	ATM	[120,121,122]
Rift Valley fever virus	RVFV	ssRNA, arbovirus	ATM, CHK2, H2AX, p53	ATM, CHK2, p53	[123,124]
Hepatitis C virus	HCV	ssRNA, flavivirus	ATM, CHK2, H2AX, CHK1	ATM, CHK2	[125,126]

HSV-1, an alphaherpesvirus, has a complex relationship with the DDR, in that it activates many components of the ATM-dependent signaling pathway, such as phosphorylation of CHK2, 53BP1, and NBS1, while inhibiting the DNA-PKcs and ATR kinases [95,127,128,129]. MRE11-RAD40-NBS1 (MRN) complex formation (Figure 1) and activated ATM promote HSV-1 replication [95]. HSV-1 codes for an immediate early transcription factor, ICP0 that promotes cell cycle arrest by inducing the tumor suppressor p53 and its downstream target proteins (p21, GADD45, and MDM2).

Epstein-Barr virus (EBV) is a gammaherpesvirus that induces the phosphorylation of ATM, NBS1, H2AX, CHK2, and p53 during lytic infection [99]. Phosphorylated ATM, NBS1 and Mre11 proteins are recruited to EBV replication compartments. XPC, a sensor of DDR that functions in nucleotide excision repair, is required for EBV replication [96].

The murine gammaherpesvirus 68 (γHV68) latency-associated, anti-interferon M2 protein induces ATM activation and histone acetylation [100] presumably to limit the induction of a virus-induced DNA damage signaling cascade. However, γHV68 protein kinase orf36 activates the DDR and facilitates lytic replication in primary macrophages. H2AX, an orf36 substrate, can enhance MHV68 replication [101].

The large T antigen encoded by the SV40 polyomavirus, deregulates multiple DNA damage signaling and repair pathways [97]. ATM mediated phosphorylation of SV40 large T antigen is detected at the onset of viral replication, and is required for optimal viral DNA synthesis [102]. Inhibition of ATM activity decreases SV40 DNA accumulation [102,103], and delays the assembly of viral replication compartments and recruitment of cellular DNA repair proteins to these sites.

Human papillomavirus (HPV) proteins induce a DDR characterized by the activation of the ATM kinase substrates CHK2, NBS1, and BRCA1 [104,105,106,107,108]. ATM kinase activity is required for HPV genome amplification in differentiating cells but not for episome maintenance in undifferentiated cells [106]. HPV does not induce degradation of MRN components but instead keeps them at high levels throughout differentiation [106]. 

As a single stranded DNA virus, human parvovirus B19 (B19V) induces a broad range of DDR by triggering activation of all PI-3-like kinases associated with DNA repair pathways during infection [109,110,111]. Phosphorylated ATM, ATR, and DNA-PKcs, and their downstream targets (CHK2, CHK1, and Ku70/Ku80 complex, respectively) are all localized within B19V replication compartments. However, B19 virus apparently only uses ATR-CHK1 signaling to promote its replication [111].

Relatedly, adeno-associated viruses (AAV), another member of Parvoviridae, do not have an absolute requirement for ATM kinase activity. DNA-PK is the primary mediator of damage signaling in response to AAV replication [113]. Immunofluorescence revealed that some activated damage proteins are found in a pan-nuclear pattern (phosphorylated ATM, SMC1, and H2AX), while others such as DNA-PK components (DNA-PKcs, Ku70, and Ku86) and RPA32 accumulate at AAV replication compartments. DNA-PK enhances recombinant AAV (rAAV) replication through the interaction of Ku proteins and AAV-ITRs [112].

ATM protein can also enhance the replication of retroviruses and lentiviruses [120,121], such as human immunodeficiency virus type 1 (HIV-1) where ATM can enhance HIV replication by stimulating Rev function [120,122]. Similar observations have been made during human T‑cell lymphotropic virus (HTLV1) infections [114,115,116,117,118,119]. 

In most cases, ATM signaling has been demonstrated to be beneficial for DNA virus replication. It has also been suggested that RNA viruses activate DDR functions that can be beneficial. A study shows the induction of DNA damage signaling upon infection with Rift Valley Fever Virus (RVFV), an RNA virus, that results in cell cycle arrest and increased viral replication [124]. ATM and a number of its substrates, CHK2, H2AX, and p53, were phosphorylated following RVFV infection. The use of ATM and CHK2 inhibitors or *p53*-null cells demonstrates that they are required for RVFV replication [123,124]. Another recently identified example of an RNA virus that activates DDR is HCV, which replicates better in the presence of ATM and CHK2, and expresses viral proteins that bind ATM and sensitize cells to DNA damage [125,126]. Other studies observe that HCV NS2 protein inhibits DNA damage signaling by sequestering p53 in the cytoplasm while the viral core protein interacts with NBS1 protein, leading to inhibition of MRN complex formation thereby blocking ATM activation and signaling in response to DSBs [130,131]. Clearly the association between RNA virus infection and DDR is complex, with additional study needed to better understand the molecular underpinnings and biology of this relationship.

In contrast, some viruses do not use DDR signaling for replication [91]. Specially, serotype-specific inactivation of the cellular DDR during adenovirus infection has been found [132]. For example, human adenovirus serotype 5 (Ad5) encoded E4 proteins inactivate the MRN complex early in infection, either via E1b55K/E4orf6-mediated degradation of MRN [90,133] or E4orf3-mediated mislocalization of MRN into nuclear tracks [134,135] and cytoplasmic aggresomes [136,137]. In addition to preventing ATM and ATR-mediated damage signaling, inactivation of MRN promotes Ad5 DNA replication [134,138,139]. Likewise, Kaposi’s sarcoma-associated herpesvirus (KSHV) viral interferon regulatory factor 1 (vIRF1) compromises an ATM/p53-mediated DNA damage checkpoint by targeting both upstream ATM kinase and downstream p53 tumor suppressor [140]. Whether this activity is essential for productive or latent infection awaits further study.

In summary, though there are some viruses that do not use DDR for their replication (*i.e.*, Ad5), there are numerous viruses (Table 1) that induce DDR pathways and require ATM or other PI-3-like kinases for productive infection. For more detailed information of DDR and viruses in general, see the following reviews [85,86,87,88,89]. 

## 8. HCMV Modulates the Cell Cycle and Checkpoints

**Figure 2:**
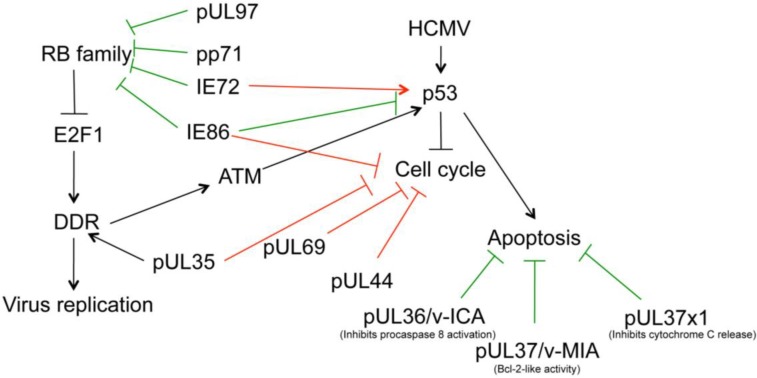
The relationship between cell cycle and the DDR induced by HCMV. Lines depicted in green represent activities that promote cell cycle progression or prevent the cells from undergoing apoptosis. Lines depicted in red represent activities that can negatively affect cell cycle progress within the cell.

**Figure 3:**
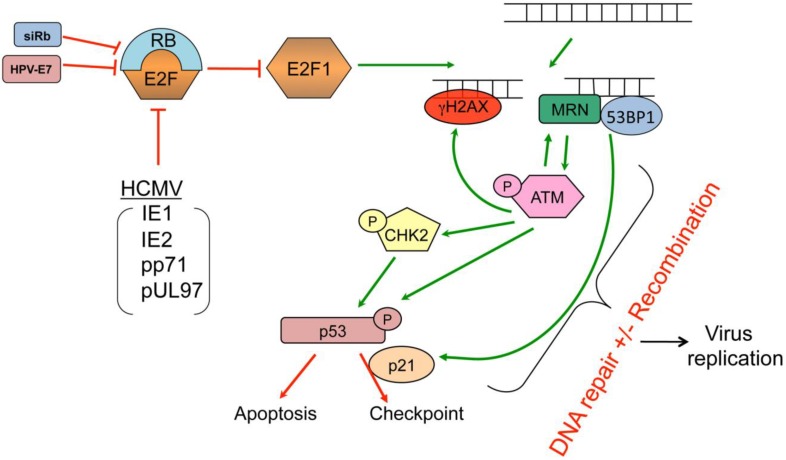
Model of the host DNA damage response induced by HCMV infection.

HCMV infection can alter the cell cycle status (Figure 2) and induce a DDR (Figure 3). Most cells infected with HCMV are driven into a unique G1/S-like state that provides enzymes and metabolites necessary for viral DNA replication [141,142,143]. At the same time, the virus directly suppresses competitive cellular DNA synthesis [144,145,146,147]. This unusual G1/S-like state is dependent upon several viral proteins including at least the tegument proteins pUL69 [148], pp71 [149], and pUL97 [150], pUL35 [151], the immediate early proteins, IE1 [152] and IE2 protein [153], and polymerase accessory protein pUL44 [154], while several other viral proteins prevent apoptosis signaling that would normally result from deregulating the cell cycle (Figure 2). 

The p53 checkpoint protein functions primarily as a transcriptional activator with target genes including the CDK inhibitor, p21, as well as Mdm2, a negative regulator of p53. The levels of p53 and its phosphorylation are increased during HCMV infection [94,98,141]. Phosphorylation-mediated activation of p53 induces an arrest of the cell cycle, which is mainly due to the induction of p21, sustained arrest leading to apoptosis. During HCMV infection, phosphorylation of p53 following DDR signaling prevents its interaction with MDM2, thereby stabilizing p53 by preventing its ubiquitin-mediated degradation [155], and contributing to the G1/S-like cell cycle arrest through the induction of p21 expression [152]. HCMV infection also activates several factors that normally induce cell cycle progression, including E2Fs [156]. In summary, the interaction between HCMV and cell cycle regulatory mechanisms is complex, with some viral factors eliciting cell cycle arrest and others promoting cell cycle progression. Thus, HCMV infection-induces a cell cycle arrest that leaves cells in a unique state that favors its replication, a theme that appears to be common to herpesviruses during productive infections [92]. 

## 9. HCMV, Deregulation of the Cell Cycle, and DNA Damage Signaling

HCMV infection induces a DDR that includes activation of ATM, H2AX, NBS1, CHK2, CHK1, and p53 [94,98,157]. How does HCMV induce DDR signaling? HCMV encode four proteins, IE1, IE2, pp71 and pUL97 that can either bind to or phosphorylate RB family members (Figure 2) [17,149,150,158,159,160,161,162,163]. Binding by these viral proteins to or phosphorylation of pRB causes the release of E2F proteins. Of these, the now de-repressed E2F1 can induce DSBs [164]. The resultant ATM activation and its downstream phosphorylation targets, including H2AX and p53, contribute to HCMV replication [94]. These DDR phenotypes are similar to productive infection with another herpesvirus, HSV-1 [85,89,95,128]. In addition, HCMV pUL35 can active DDR, causing γH2AX and 53BP1 foci formation and induce a cell cycle arrest [151] which likely supports viral replication [151]. 

HCMV, in particular, encodes several proteins that both modulate cell cycle controls and the host DDR to promote viral replication. The following subsections focus on how some of these viral proteins, in particular, IE1, IE2, pp71, pUL97, pUL69, mediate these activities.

### 9.1. IE1 Can Inactivate p107 and p130

*UL123*, encoding IE1 (sometimes referred to IE1-72 or IE72), is the first transcribed HCMV gene. As a predominantly nuclear protein, IE1 is a promiscuous transactivator that also interacts and modulates the function of p107, a member of the RB protein family [17,165]. IE1 has been suggested to exhibit kinase activity [156,163]. *In vitro* kinase assays suggest that both p107 and p130 can be phosphorylated by IE1 and that phosphorylation of these two RB proteins is sufficient to disrupt their interaction with E2F4 [163]. These findings imply that IE1 specifically targets at least two RB proteins for inactivation, which causes the derepression of E2F-responsive promoters. Even with RB family member inactivation and derepression of E2Fs, IE1 expression is unable to induce S-phase entry in primary fibroblasts due to a p53-dependent arrest [152]. This ability of p53 to block the IE1-mediated induction of S-phase is dependent on the p21 CDK inhibitor [157]. 

IE1 also disrupts PML bodies [166,167,168] (also known as promyelocytic oncogenic domains (PODs) or nuclear domains-10 (ND-10)). PML is involved in cellular growth regulation, transcription, DNA replication and repair, and posttranscriptional regulation of gene expression [169]. PML body integrity is also a component of the host intrinsic antiviral defense and the displacement of PML protein from PML bodies by IE1 contributes for sustained viral gene expression [170,171].

### 9.2. IE2 Binds pRB and p53

Studies have shown that the protein encoded by the largest transcript of *UL122*, IE2 (also known as IE2-86 or IE86) specifically interacts with pRB [16,160,172,173]. Expression of IE2 is sufficient to alleviate pRB repression of E2F-responsive promoters [16,47,160]. IE2 can also interact with p53 [174,175,176]. However, expression of IE2 in a human cell line blocked cell cycle progression in G1-phase similar to the phenotype observed when permissive cells are infected with HCMV [153]. Moreover, a study using DNA microarrays to analyze the effects of IE2 protein on cellular gene expression reveals that the 86kDa form of IE2 induces the expression of numerous factors associated with cell cycle regulation and bioenzymatic machinery necessary for DNA replication. For example, IE2 expression results in an increase in the mRNA levels of B-myb, cyclin E, cdk-2, E2F1, ribonucleotide reductase subunit-1 and -2, thymidine synthetase, MCM3, and MCM7, among other factors associated with S phase [177]. This study shows that most of the genes induced by IE2 are E2F targets. What is striking is that even though IE2 inactivates pRB, derepresses E2F activity, and can inhibit cell cycle arrest functions of p53, this viral protein does not induce S phase as measured by cellular DNA replication. This observation is in stark contrast to pRB and p53 targeting proteins encoded by other viruses such as SV40 lg T or the combination of adenovirus E1A and E1B-55K proteins, which are potent inducers of cell cycle progression and S phase. A clearer understanding is needed as to why IE2 does not induce S phase.

### 9.3. pp71 Binds to pRB, p107 and p130

The pp71 phosphoprotein, expressed from the *UL82* ORF, is a tegument protein that localizes to the nucleus immediately after virion entry during productive infections [178,179]. Functionally, pp71 is a transcription factor that is packaged within the viral tegument and is essential for the adequate accumulation of IE1 and IE2 by transactivating the major IE promoter and accelerating the infection cycle of HCMV [180]. Studies examining the relationship between pp71 and the cell cycle reveal that pp71 contains a sequence (LACSD) that is similar to the RB-binding motif (LxCxE) present in viral oncoproteins encoded by the small DNA tumor viruses. This motif is required for the induction of DNA synthesis in quiescent cells and for degradation of the RB family by viral oncoproteins [149,162]. pp71 binds to all three RB family members and promotes the G1/S cell cycle state during infection [149].

### 9.4. pUL97 Phosphorylates RB Protein

pUL97, another tegument protein, is a multifunctional viral protein kinase which is required at multiple steps during viral replication. Deletion of the pUL97 region from the viral genome or pharmacological inhibition of pUL97 kinase activity drastically reduces viral replication. A number of cellular and viral interacting proteins and substrates of pUL97 have been described, including viral pUL69 [181], pUL44 [182], autophosphorylated pUL97 [183,184] , and, given its cdk-like activity, cellular RB family proteins are also phosphorylated by pUL97 [150]. In addition, pUL97 is able to phosphorylate nuclear lamins, which contributes to the HCMV-induced reorganization of the nuclear lamina [185]. 

### 9.5. pUL69 Modulates CDK Function

As mentioned earlier, IE1, IE2 and pUL97 can interact or phosphorylate RB family members to inactivate protein function, which can, under certain circumstances, result in stimulation of cell cycle progression. However, during HCMV infection, cell cycle progression is blocked at multiple points, including the G_1_-to-S-phase transition. pUL69, a phosphorylated virion tegument protein [186], is reported to be a factor responsible for this cell cycle block [148]. Host CDKs and pUL97 phosphorylate pUL69 and modulate its nuclear localization and activity [181]. Although the mechanism by which pUL69 induces an accumulation of cells in G1 is not clear, these findings indicate that HCMV tegument proteins (pUL69, pp71, and pUL97) can have an immediate effect on the cell cycle. It seems that HCMV has developed a strategy to inhibit cellular DNA replication and, at the same time, alter the host environment in a manner that ensures the replication of viral DNA.

### 9.6. E2F1-mediated DNA Damage Response

In the normal condition, RB family members bind to E2F family members to limit cell cycle progression. During HCMV infection, at least four viral proteins (IE1, IE2, pp71 and pUL97) inactivate RB family proteins resulting in the release of E2F proteins. These derepressed E2F proteins then alter the expression of S phase genes that contribute activities and substrates that support viral DNA replication. In addition, deregulated E2F1 induces DSBs and stimulates a robust host DDR [94]. The mechanism responsible for this phenomenon is unclear but it is specific to E2F1 as other RB-targeted E2Fs, namely E2F2 or E2F3, do not induce double strand DNA breaks (DSBs) or DDR [164]. This relationship between E2F1 and DSBs appears relevant to HCMV, as it is the only activator E2F that contributes significantly to viral replication [94]. Moreover, ATM, the signal transducing kinase of many DDR, functions downstream of E2F1 deregulation and is also required for HCMV replication [94]. 

## 10. Which DDR Factors Contribute to HCMV Replication?

ATM has been implicated as a target of several DNA viruses (Table 1), which activate or inhibit the ATM signaling pathway [85]. Depletion or inhibition of ATM by RNA interference or by pharmacological compounds, respectively, demonstrate that ATM is a key kinase in DDR and also the most common DDR factor contributing to virus replication [94]. ATM and at least some downstream targets, like H2AX [94], p53 [187] contribute to HCMV replication. In addition, at least one DNA repair factor, DDB2 [188], influences HCMV replication.

## 11. Difference and Similarities in the DDR Induced by HCMV and Other Viruses

### 11.1. HCMV Is Similar to Other Viruses that Use DDR (ATM Signaling) for Replication

As listed in Table 1, many viruses can activate ATM, and most of them can use ATM signaling to promote viral replication [94,95,97,99,100,101,102,103,104,106,108,109,110,113,114,116,120,122,124,125]. HCMV infection as well as IE1 or IE2 transduction can activate ATM [94,98,157]. Using caffeine, a PI3 kinase-like inhibitor, KU 55933, an ATM-specific inhibitor [189], AT cells derived from patients with ataxia telangiectasia, or siRNAs to deplete ATM, results in reduced or blocked HCMV replication [94]. Here, HCMV is similar to many other viruses with DNA stages in their replication strategy, such as HSV-1, HIV, MHV-68, SV-40, and HPV, in its requirement for ATM signaling for replication.

### 11.2. HCMV is Different from Other Viruses Not Using ATM or that Block DDR for Replication

HCMV is different from Ad5 and KSHV or B19V in its requirement for ATM signaling for efficient replication. Ad5 has evolved mechanisms to inhibit DNA damage signaling and repair during infection by degrading and mislocalizing components of the Mre11–Rad50–NBS1 (MRN) DNA damage recognition complex [135]. In addition, the Ad5 E3 ligase complex (comprised of E1B-55K and E4 adenoviral proteins) is able to target a number of cellular DNA repair proteins for proteasomal degradation including the RecQ helicase, bloom helicase (BLM) [190]. KSHV vIRF1 protein compromises an ATM/p53-mediated DDR by targeting both upstream ATM kinase activity and also downstream p53 tumor suppressor function by facilitating its proteasome-mediated degradation [140]. However, a recent study shows that during early *de novo* infection of primary endothelial cells, KSHV induces DDR signaling and ATM kinase activation as measured by H2AX phosphorylation, which are essential for KSHV’s latent gene expression and establishment of latency [191]. Taken together, it seems that KSHV is able to both inhibit, and induce the cellular DDR dependent upon its replication strategy. Infection with B19V induces a broad range of DNA damage responses by activating three upstream kinases: ATM, ATR, and DNA-PKcs. Disruption of either the ATR or DNA-PKcs, but not ATM, signaling pathways significantly reduced the efficiency of B19V replication without affecting the resultant cell cycle arrest [111]. Thus, it appears that B19V uses ATR and DNAPKcs, but not ATM to facilitate its replication. Likewise, DNA-PKcs contribute to the replication of adeno-associated virus (AAV), another parvovirus [112]. Interestingly, AAV and Ad5 coinfections activate a broad DDR that is different from that seen during Ad5 or AAV infection alone [113].

## 12. HCMV Infection Results in the Relocalization of DDR Proteins to Virus Replication Compartments (RCs)

Viral RCs are sites of viral DNA replication and maturation. RCs begin as multiple, discrete structures early in infection, then move and coalesce into a single, large structure that can be referred to as a “mature” RC [94,192]. HCMV DNA replication happens in the RCs, similar to HSV-1 [95,193,194]. During HCMV infection, many DDR proteins are relocalized to the RCs including γH2AX, p53, pATM, MRE11, CHK2, NBS1, Rad50, ATRIP, and CHK1 [94,98]. Although the mechanism(s) responsible for this relocation is unclear, these DDR proteins might interact with the viral DNA replication machinery in RCs to regulate viral DNA replication, gene expression, or recruit DNA repair proteins to repair damage to viral DNA.

## 13. Does DNA Damage Exist in Cellular or Viral DNA During Infection?

Many DNA viruses and retroviruses can tether or integrate their genome into the host DNA during infection [195,196]. It has been suggested that viral genetic material can be recognized by the host as damage DNA and stimulate cellular DNA repair mechanisms [197]. Given these observations, questions arise regarding whether host DNA is damaged during infections and whether DNA damage exists in viral DNA. 

Host chromosome breaks have been observed in HCMV-infected cells. Infection of cells during S-phase results in two specific breaks on chromosome 1 at positions 1q42 and 1q21 [198]. HCMV infection-associated damage to chromosome 1 appears not to be cell-type-or-strain specific. Cells infected with HSV also show an increased incidence of chromosome breaks [199,200]. Chromatid gaps and breaks were found to accumulate in region 3 of the X chromosome and in region 7 of chromosome 1 [201]. Adenovirus type 12 E1B protein can induce damage specifically at 17q21–22, lp36, 1q21, and lq42-43 and at random sites in cellular chromosomes [202]. For HCMV, it is not clear if infection causes extensive host DNA damage or whether the two DNA break is sufficient to initiate the observed host DDR.

Some studies suggest that viral adsorption and penetration is required for inducing chromosomal breaks whereas viral protein expression is not required for induction of damage [198]. Alternately, a viral or cellular protein component of the incoming virion may be responsible for the induced damage. Our laboratory has observed DDR at very early times of infection, even before *de novo* viral protein expression. Input tegument proteins have been suggested to be partly responsible for DDR soon after viral entry, perhaps by inactivating pRB and derepressing E2F1 [199]. 

Another possible source of the DDR is that incoming, virion-delivered viral DNA or the nascent viral DNA generated during replication, is damaged or contains mutations. Evidence for these possibilities exists for HSV [194,200,201,202,203,204]. One can imagine that the oxidative state of virally infected cells, in particular HCMV infected cells [205] may result in oxidation of nucleotides such as guanines in viral DNA. It is also possible that, even though herpesviruses encode replicase complexes with proof reading activity, the high levels of (viral) DNA replication during infection may result in an accumulation of mutations that stimulate the relocalization of DDR proteins to viral RCs. 

Activation of DDR does not necessarily require DNA lesions. Prolonged binding of DNA repair factors to chromatin can elicit DNA damage response in an ATM- and DNA-PK dependent manner in the absence of DNA lesions [206]. The herpesviral genomes are synthesized in a rolling circle manner to produce head-to-tail concatemers that are subsequently cleaved into unit-length genomes [207] and either the replication complexes or the cleaved DNA may be recognized as damaged DNA and trigger a DDR. In addition, viral infections confront cells with large amounts of exogenous genetic material that might be broadly recognized as abnormal [197] or the physical interaction of DNA repair factors with chromatin can be sufficient to activate the DDR signaling cascade [206]. Another possibility is that infected cells recognize viral replication as a genotoxic stress and elicit a DDR. In the case of HCMV infection, the inactivation of RB family members by IE1, IE2, pp71, and pUL97 and subsequent deregulation of E2F1 appears to result in DSBs in human fibroblasts [94]. Thus, it is possible that a trigger of the virus-induced DDR is not necessarily the recognition of linear viral DNA as double-strand breaks or actual damage to DNA, but rather, it is the recruitment of DNA damage repair factors to RCs or, very likely, a combination of these possibilities. Clearly, much work is needed to understand the interplay of viral infection, the presence of viral DNA and proteins, the remodeling of host cells function and the resultant DDR.

## 14. Future Perspectives

Although it is well accepted that activation of host DDR is a common theme of infections with DNA viruses, many of which require DDR signaling to replicate, more detailed study is still needed to better understand the “hows” and “whys” of this relationship. Most small DNA viruses capable of infecting nondividing cells induce S phase in order to activate the host DNA replication machinery to provide the nucleotide triphosphates and host replication machinery necessary for viral DNA replication [93]. However, many large DNA viruses, such as herpesviruses, code for not only their own viral replicase enzymes, but also factors involved in deoxynucleotide synthesis and do not require a canonical S phase to support viral replication [92]. It has been thought that the host DNA polymerases do not play a role in herpesviral DNA replication. However, our unpublished studies suggest that host DNA polymerases may contribute to HCMV replication with the implication that theses enzymes may contribute activities that are different from the viral polymerase [208]. It should be interesting to explore the roles of host DNA polymerases in the replication of HCMV and other viruses.

Components of the ATM-pathway are activated and recruited to sites of viral DNA synthesis including HCMV infected cells. Although ATR- and ATM-mediated pathways are related and both can be activated by similar genotoxic events, HSV-1 distinguishes between these two pathways, inactivating ATR pathway and potentially using ATM pathway. In contrast, it has been noted that the steady-state levels of ATR increase in HCMV infected cells at 48 hpi accompanied by a shift in mobility [98]. The ATR pathway also responds to DSBs, generally more slowly than ATM [209]. Determining the contribution of ATR signaling in relation to ATM signaling during HCMV infection should provide important insight into the specifics of DDR signaling as it pertains to HCMV infection and viral DNA replication,

HSV and HCMV infections, and the adenovirus type 12 E1B protein induce chromosome breaks in specific chromosome regions. The particular nonrandom distribution of chromosome aberrations in the HSV and HCMV infected cells raise a question as to whether virion genome deposition or early replication localized to particular chromosome regions are responsible for these effects.

The mechanism by which E2F1 stimulates host DDR is not well understood. Inactivation of RB and the subsequent deregulation of E2F1, but not the related family members, E2F2 or E2F3, leads to an accumulation of DSBs in human fibroblasts as observed by the γH2AX immunostaining and neutral comet assays [164]. The mechanism by which E2F1 stimulates host DDR needs to be further studied during infection by HCMV and other viruses.

Ubiquitination and sumolyation is one of the most common mechanisms by which viruses target cellular proteins. Viruses can encode their own ubiquitin ligases, such as the ICP0 protein of HSV-1. ICP0 interacts with PML isoform I and induces its SUMO-independent degradation [210]. Other viral proteins, such as Ad-E1B55K/E4orf6, KSHV-LANA, HPV-E6, and HIV-Vpr, can recruit and redirect cellular ubiquitin ligase complexes to target proteins. A recent study shows that a DNA repair factor, DDB2, can contribute to HCMV replication [188]. DDB2 is a component of a Cul4A-Ub ligase complex. The DDB1-CUL4A^DDB2^ complex is a cullin-RING (*i.e.*, E3) Ub-ligase that targets histone H2A at UV-damaged DNA sites. Whether the ubiquitination ability of this complex is involved in HCMV replication needs further investigation.

One wonders if the intimate relationship between the host DDR and HCMV infection can be leveraged to treat HCMV-associated diseases. For example, there are no licensed treatments for pregnant women who undergo primary infection with HCMV during pregnancy, which places the fetus at risk for symptomatic congenital infection. This is at least partly due to the fact that drugs that are effective against HCMV infection have serious teratogenic side effects, which make them inappropriate for use during pregnancy. HCMV is also a common complication during tissue transplantation. Here, ganciclovir is commonly used to treat HCMV disease. However, ganciclovir induces leukocytopenia, an unwanted side effect in patients already receiving immunosuppressants. Perhaps, ATM inhibitors or more appropriately, drugs that target ATM responsive factors can be used alone or as adjuvant therapies to prevent congenital CMV infections or other diseases associated with infection. Continued study of infection-induced DDR pathways is essential for a better understanding of host-pathogen interactions and the development of host-targeted therapeutics.
